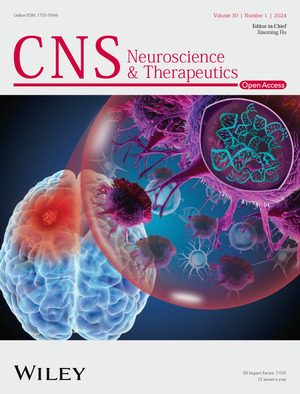# Additional Cover

**DOI:** 10.1111/cns.14732

**Published:** 2024-05-02

## Abstract

The cover image is based on the Original Article *PVT1 promotes proliferation and macrophage immunosuppressive polarization through STAT1 and CX3CL1 regulation in glioblastoma multiforme* by Lijie Huang et al., https://doi.org/10.1111/cns.14566.